# Echocardiographic hemodynamics correlate with differences in DOAC-specific bleeding and stroke rates in non-valvular atrial fibrillation

**DOI:** 10.1186/s12872-025-05239-w

**Published:** 2025-10-28

**Authors:** Michael P. O’Shea, Ali Yusuf, Eiad Habib, Srekar Ravi, Suganya Arunachalam Karikalan, Chieh Ju Chao, Hasan Ashraf, Pradyumna Agasthi, Sam Newton, Robert L. Scott, Timothy Barry, Chadi Ayoub, Reza Arsanjani, Hicham El Masry

**Affiliations:** 1 Mayo Clinic, 134000 E Shea Blvd, Scottsdale, AZ 85259 USA; 2 London School of Hygiene and Tropical Medicine, London, England; 3 Mayo Clinic, Rochester, MN USA; 4 Stanford University, Palo Alto, CA USA; 5 Christus Good Shepherd Heart and Vascular Institute, Longview, TX USA; 6 Sanford Health, University of North Dakota, Sanford, Florida USA; 7 Kwame Nkrumah University of Science and Technology, SCMC01IMEDUC, Kumasi, Ghana

**Keywords:** Anticoagulation, Atrial Fibrillation, DOAC, Bleeding, Pulmonary Hypertension, Right ventricular systolic pressure, Apixaban, Rivaroxaban, Dabigatran

## Abstract

**Aims:**

Direct oral anticoagulants (DOACs) are widely used for stroke prevention in people with non-valvular atrial fibrillation/flutter (NVAF).Anticoagulants have different bleeding profiles. Apixaban is associated with the lowest bleeding risk. This study evaluates the interaction between DOAC use and right ventricular systolic pressure (RVSP) on echocardiogram and bleeding rates. It was hypothesized that elevated RVSP may be associated with excess bleeding based on anticoagulant-specific pharmacologic profiles.

**Methods& Results:**

A retrospective analysis of a database was conducted. Multivariable regression models for bleeding rates were developed using an analysis approach that adjusts for confounders among participants who had undergone an echocardiogram, and interaction terms for DOAC choice were incorporated. Stratum-specific estimates were calculated using linear computation.

Patients taking apixaban had lower bleeding rates than those taking rivaroxaban and dabigatran. Among those without elevated RVSP, there was no difference in bleeding rates. Where RVSP was ≥45mmHg, there was a 90% increase in bleeding rates with use of dabigatran (HR 1.99, p=0.024) and rivaroxaban (HR 1.86, p=0.042) compared to apixaban (Table 1D).

**Conclusions:**

Elevated RVSP is associated with excess bleeding in patients taking rivaroxaban and dabigatran, but not apixaban, after controlling for confounding variables.

**Supplementary Information:**

The online version contains supplementary material available at 10.1186/s12872-025-05239-w.

## Introduction

Stroke is the second leading cause of death globally and the third leading cause of disability [1]. There are approximately 800,000 strokes in the US, of which 87% are ischemic [2]. Atrial fibrillation (AF) and atrial flutter (AFL) are supraventricular arrhythmias that result in a 2-to-5-fold increase in ischemic stroke risk, as initially established in the Framingham Heart Study [3–8]. Warfarin, a highly effective stroke prevention therapy, quickly became standard of care, despite requiring frequent drug monitoring and having multiple drug interactions [9]. More recently, four direct oral anticoagulant (DOAC) alternatives to warfarin (apixaban, rivaroxaban, dabigatran, and edoxaban) were approved for stroke prevention in patients with non-valvular AF (NVAF) based on the results of non-inferiority trials of varying quality [10–13]. They have become the preferred choice over the last decade [14].

Warfarin and DOACs each have unique bleeding profiles. All DOACs have a relatively lower risk of intracranial hemorrhage [15–17]. Apixaban has the lowest rate of bleeding among DOACs, and this could be explained by its uniquely lower rate of GI bleeding compared to warfarin [15, 18–21]. Given that all DOACs demonstrated similar non-inferiority in clinical trial settings compared to warfarin, real-world differences in patient characteristics and comorbidities should reasonably explain reduced bleeding rates with apixaban compared to other drugs. Little is known about factors that modify the association between DOAC choice and bleeding rates.

Heart failure is associated with altered oral drug absorption and metabolism [22]. Pharmacokinetics of many drugs are modified to a clinically significant extent in heart failure exacerbations, including warfarin, furosemide, theophylline, and specific ACE inhibitors [23, 24]. Right ventricular hypertension (RVH) is a feature of heart failure seen in patients with right ventricular outflow tract obstruction or, more commonly, pulmonary hypertension (PH). It is associated with elevated right ventricular systolic pressures. PH is a condition characterized by elevated pulmonary arterial pressure due to changes in the pulmonary vasculature. It is diagnosed when the mean pulmonary artery pressure is greater than 20 mmHg or the pulmonary vascular resistance is greater than 2.0 Wood units [25]. Notably, individuals managed on specific PH-directed therapies have a hypothetical risk of bioaccumulation of DOACs in their system [26]. Increased central venous pressure, a feature of PH, could also potentially contribute to a higher incidence and severity of bleeding episodes. This is supported by observational data conducted on patients with heart failure, where there is an increased risk of bleeding case fatality compared to individuals without heart failure [27].

We hypothesize that these cardiac hemodynamic measures will correlate with altered DOAC-specific stroke and bleeding events.

### Objectives

This study evaluated the association between DOAC choice and bleeding and stroke rates among a real-world cohort, stratified by ejection fraction and right ventricular systolic pressure.

## Materials and methods

### Study design

The protocol for data collection in this retrospective cohort study was previously reported [28]. The exposure was the DOAC choice. The primary outcomes were time to first major bleed on a DOAC, as defined by the International Society of Thrombosis and Hemostasis [29]. Secondary outcomes were bleeding subcategories of intracranial and gastrointestinal hemorrhage, and time to first stroke. Participants entered the study at the time of DOAC initiation. Participants exited the study at the last follow-up or were censored after experiencing an event. Participants who had not undergone a transthoracic echocardiogram were excluded from analysis.

### Study participants

Patients with NVAF who were receiving a DOAC and who attended any of the Mayo Clinic Sites (AZ, FLA, MN) or the Mayo Clinic Health System on two separate occasions between January 2001 and December 2017 were included in the study. Exclusion criteria were:

- Anticoagulation exclusively for venous thromboembolism.
- History of intracardiac thrombus or heparin induced thrombocytopenia.
- Moderate or severe mitral stenosis.
- Rheumatic heart disease.
- End-stage renal disease requiring dialysis or creatinine clearance < 15mL/min.
- DOAC discontinuation or switching to a different anticoagulant during follow-up.
- Follow-up for less than 30 days.
- Use of edoxaban (due to data sparsity).
- No documented creatinine clearance.
- CHADS-VASC score < 2.

### Ethics approval

Institutional approval was granted by the Mayo Clinic Institutional Review Board (Ref 19–004887) and by the London School of Hygiene and Tropical Medicine MSc Research Ethics Committee (Ref 28981). This study was conducted in accordance with the Declaration of Helsinki.

### Variables

Potential confounders included sex, age, DOAC dose, atherosclerotic disease, atrial flutter, paroxysmal atrial fibrillation, creatinine clearance (CrCl), chronic obstructive pulmonary disease (COPD), hypertension (HTN), hyperlipidemia (HLD), diabetes mellitus (DM), history of hemorrhagic stroke, history of gastrointestinal bleeding, prior ischemic stroke or transient ischemic attack (TIA), race, weight category, prosthetic valve, and left ventricular ejection fraction (LVEF).

CrCl, calculated using the Cockroft-Gault Equation, was reported based on KDIGO staging for chronic kidney disease (CKD) [30]. LVEF, calculated using Simpson’s Biplane method on transthoracic echocardiogram (TTE) imaging, was stratified to ≤ 40%, 41–49%, and ≥ 50% based on European Heart Association diagnosis categories [31–34]. Right ventricular systolic pressure (RVSP), calculated using the modified Bernoulli Equation plus the estimated right atrial pressure, was categorized as normal (0 to 34 mmHg), moderately elevated (35 to 44 mmHg), and high (≥ 45 mmHg) [35]. These thresholds were selected to allow assessment of biologic gradient (a component of the Bradford Hill Criteria for causal association), and to avoid data sparsity in multivariable regression with interaction testing [36]. Weight category thresholds were selected to represent the 10th and 90th percentiles for this cohort. Lexis expansion was conducted to generate a term for the current age in strata of 5-year intervals to identify the bleeding rate by age category. In multivariable analysis, current age was controlled by setting the date of birth as the point of origin in Cox regression.

The primary outcome was the rate of first bleeding event following DOAC initiation in patients with NVAF. Secondary outcomes included the rate of first GI bleed following DOAC initiation, the rate of first intracranial bleed following DOAC initiation, and the rate of first stroke following DOAC initiation. Stroke rate analysis was restricted to participants who had never had a prior stroke before DOAC initiation.

### Statistical analysis

The association between DOAC choice and clinical characteristics were assessed using odds ratios and chi-squared test for heterogeneity. The association between variables and outcome variables was calculated using the rate ratio and the chi-squared test. Confounding between DOAC choice and bleeding rate was assessed using bivariable regression, controlling for each variable in turn and controlling for current age. A Cox multivariable regression model incorporating the confounders identified above, in addition to a priori confounders of age and sex, was developed [37]. Interaction parameters between DOAC choice and RVSP were included, and stratum-specific estimates were calculated using linear computation (linear combination of estimators or *lincom* function on Stata) and Wald test p-values. Likelihood ratio testing for improved fit with interaction was conducted for RVSP and DOAC choice.

Predicted bleeding rates were calculated by multiplying the estimated bleeding rate for each individual based on the ORBIT score bleeding group category (low, medium, or high) by the individual’s time in the study [38]. The percentage of bleeds predicted was calculated using the actual and predicted number of bleeds in each stratum of RVSP. A similar approach was taken with stroke risk using individual CHA_2_DS-_2_VASc score stroke estimates calculated from the Swedish Atrial Fibrillation Cohort Study [39].

A similar analysis was conducted for secondary outcomes, with modifications made to account for data sparsity. Specifically, for intracranial bleed, confounders were not incorporated due to outcome sparsity. For analysis of the rate of stroke following DOAC initiation, the analysis was restricted to people who had no prior stroke. Analysis was conducted using Stata/IC 16.1.

## Results

**Fig. 1:**
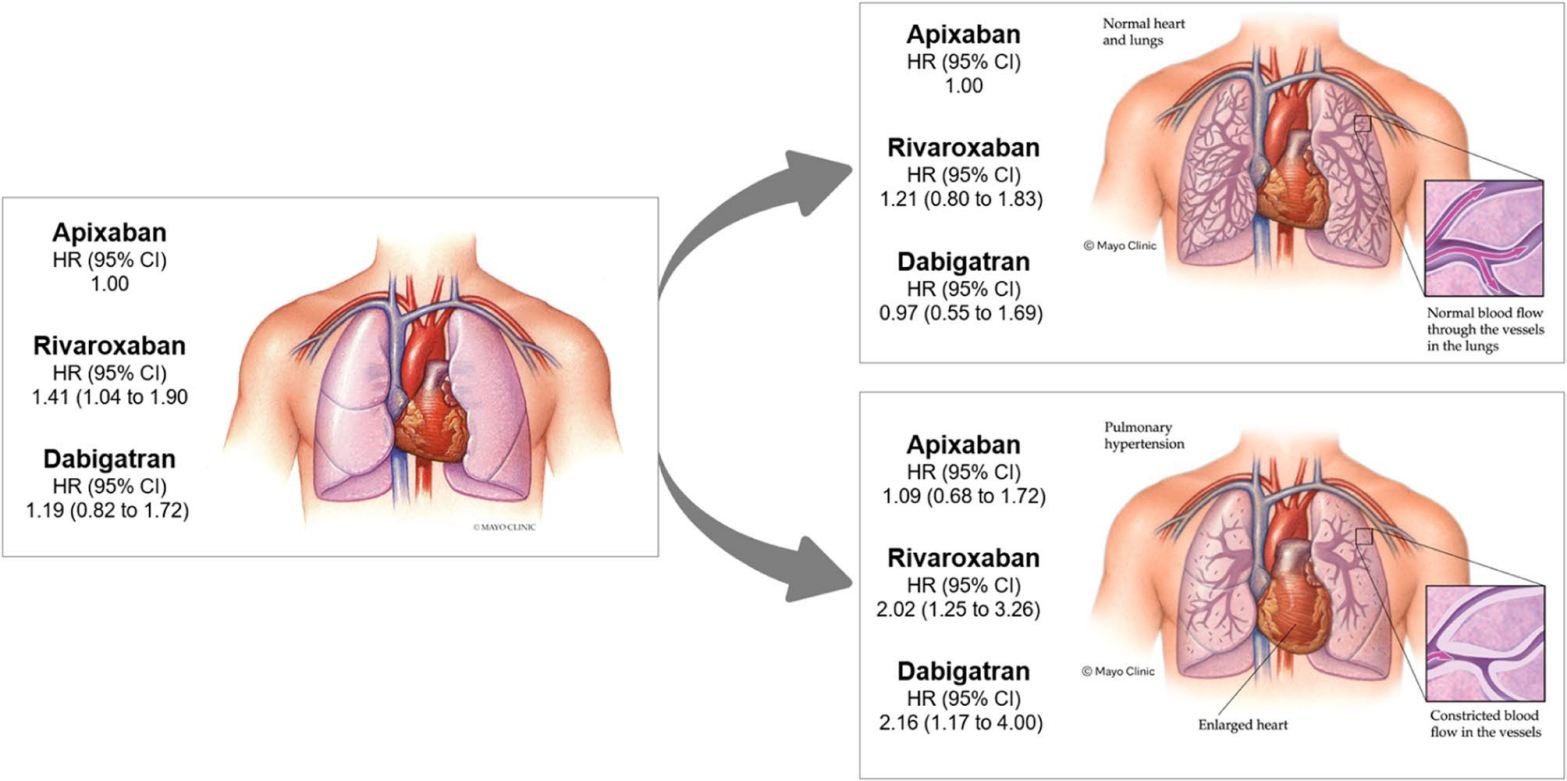
Participant inclusion and exclusion flowchart

A total of 8,125 patients were identified as having NVAF and were on DOAC therapy. After applying the exclusion criteria, 2,308 participants were included in the final study (Fig. 1). For analysis of stroke rates only, an additional 465 patients were excluded for having had a prior stroke.

Variables which were associated with DOAC choice included sex, age, presence of AFL, paroxysmal AF, creatinine clearance, history of GI bleed, prior stroke or transient ischemic attack, weight category, and RVSP (Supplemental appendix (SA) Table 1).

**Table 1:** A-D: HR for bleeding rate among people with NVAF by DOAC choice, calculated using multivariable Cox regression controlling for current age and multiple confounders*. Stratum specific rates are included in a subsequent model with interaction for RVSP and DOAC choice using linear computation

**A: Estimates without interaction term**					
Apixaban		Rivaroxaban		Dabigatran	
*HR (95% CI)*	*p-value*	*HR (95% CI)*	*p-value*	*HR (95% CI)*	*p-value*
1.00		1.41 (1.04 to 1.90)	0.027	1.19 (0.82 to 1.72)	0
**B: With interaction parameters for RVSP and DOAC choice, compared to Apixaban with normal RVSP**					
Apixaban		Rivaroxaban		Dabigatran	
*HR (95% CI)*	*p-value*	*HR (95% CI)*	*p-value*	*HR (95% CI)*	*p-value*
1.00		1.21 (0.80 to 1.83)	0.367	0.97 (0.55 to 1.69)	0.901
1.13 (0.72 to 1.76)	0.597	1.55 (0.89 to 2.72)	0.125	1.13 (0.57 to 2.22)	0.730
1.09 (0.68 to 1.72)	0.728	2.02 (1.25 to 3.26)	0.004	2.16 (1.17 to 4.00)	
**C: HR examining rate changes within each DOAC with increasing RVSP**					
Apixaban		Rivaroxaban		Dabigatran	
* HR (95% CI)*	*p-value*	*HR (95% CI)*	*p-value*	*HR (95% CI)*	*p-value*
1.00		1.00		1.00	
1.13 (0.72 to 1.76)	0.597	1.28 (0.72 to 2.28)	0.396	1.17 (0.53 to 2.59)	0.703
1.09 (0.68 to 1.72)	0.728	1.67 (1.01 to 2.75)	0.044	2.24 (1.06 to 4.73)	
**D: HR examining rate changes across each stratum of increasing RVSP**					
Apixaban		Rivaroxaban		Dabigatran	
HR (95% CI)	*p-value*	*HR (95% CI)*	*p-value*	*HR (95% CI)*	*p-value*
1.00		1.21 (0.80 to 1.83)	0.367	0.97 (0.55 to 1.69)	0.901
1.00		1.38 (0.75 to 2.54)	0.306	1.00 (0.48 to 2.07)	0.999
1.00		1.86 (1.09 to 3.19)	0.024	1.99 (1.02 to 3.88)	0.042

*Variables include sex, dose indicated, CKD stage, history of GI bleed, ejection fraction, presence of atrial flutter or paroxysmal AF, diabetes, non-white race, and right ventricular systolic pressure

### Bleeding

#### Major bleeding

A total of 235 participants developed at least one major bleeding episode over 4,945.28 person-years (mean follow-up duration 2.14 years). The rate of first major bleeding event was 4.75 (4.18 to 5.40) bleeds per 100 person years. There was no evidence of difference in bleeding rates between DOAC choice on unadjusted analysis (rivaroxaban HR 1.22 [0.93 to 1.61], *p* = 0.155; dabigatran HR 1.03 [0.72 to 1.47], *p* = 0.890 compared to apixaban). Variables associated with the rate of first major bleed included age category, dose indicated, CKD stage, history of GI bleed, and RVSP (SA Table 2). Variables that altered the association between DOAC choice and bleeding rate by a cumulative absolute difference of ≥ 2% for both dabigatran and rivaroxaban compared to apixaban were identified using Cox bivariable regression. Confounders identified were sex, dose indicated, creatinine clearance, history of GI bleed, prior stroke or TIA, LVEF category, atrial flutter, paroxysmal AF, DM, and non-white ethnicity (SA Table 3).

**Table 2:** Bleeding hazard ratio for people with NVAF receiving DOACs, stratified by DOAC choice, calculated using multivariable Cox regression controlling for current age and multiple confounders*. Stratum specific estimates are included in a subsequent model with additional parameters for interaction for EF and DOAC choice, calculated using linear computation

**A: Estimates without interaction term**					
Apixaban		Rivaroxaban		Dabigatran	
*HR (95% CI)*	*p-value*	*HR (95% CI)*	*p-value*	*HR (95% CI)*	*p-value*
1.00		1.41 (1.04 to 1.90)	0.027	1.19 (0.82 to 1.72)	0.359
**B: Estimates with interaction parameters for EF category and DOAC choice**					
Ejection Fraction					
1.00		1.44 (1.03 to 2.00)	0.032	1.12 (0.73 to 1.73)	0.595
1.16 (0.60 to 2.24)	0.664	1.33 (0.63 to 2.80)	0.453	1.10 (0.34 to 3.51)	0.875
1.18 (0.66 to 2.11)	0.580	1.68 (0.84 to 3.39)	0.145	2.02 (0.97 to 4.23)	0.062

*variables include sex, dose indicated, CKD stage, history of GI bleed, ejection fraction, presence of atrial flutter or paroxysmal AF, diabetes, non-white race and right ventricular systolic pressure

**Table 3:** A-D: hazard ratios for Gastrointestinal bleeding rate among people with NVAF by DOAC choice, calculated using multivariable Cox regression controlling for current age and multiple confounders*. Stratum specific rates are included in a subsequent model with interaction for RVSP and DOAC choice using linear computation

**A: Estimates without interaction term**					
Apixaban		Rivaroxaban		Dabigatran	
*HR (95% CI)*	*p-value*	*HR (95% CI)*	*p-value*	*HR (95% CI)*	*p-value*
1.00		1.32 (0.90 to 1.92)	0.157	1.12 (0.70 to 1.81)	0.637
**B: With interaction parameters for RVSP and DOAC choice, compared to Apixaban with normal RVSP**					
Apixaban		Rivaroxaban		Dabigatran	
*HR (95% CI)*	*p-value*	*HR (95% CI)*	*p-value*	*HR (95% CI)*	*p-value*
1.00		0.91 (0.55 to 1.52)	0.720	0.79 (0.41 to 1.56)	0.504
0.60 (0.30 to 1.18)	0.137	1.42 (0.70 t0 2.86)	0.332	1.03 (0.47 to 2.26)	0.936
1.01 (0.55 to 1.85)	0.966	1.95 (1.03 to 3.70)	0.041	1.68 (0.69 to 4.07)	0.254
**C: HR examining rate changes within each DOAC with increasing RVSP**					
Apixaban		Rivaroxaban		Dabigatran	
*HR (95% CI)*	*p-value*	*HR (95% CI)*	*p-value*	*HR (95% CI)*	*p-value*
1.00		1.00		1.00	
0.60 (0.30 to 1.18)	0.137	1.55 (0.73 to 3.29)	0.248	1.30 (0.51 to 3.29)	0.581
1.01 (0.55 to 1.85)	0.966	2.14 (1.07 to 4.29)	0.032	2.11 (0.76 to 5.85)	0.152
**D: HR examining rate changes across each stratum of increasing RVSP**					
Apixaban		Rivaroxaban		Dabigatran	
*HR (95% CI)*	*p-value*	*HR (95% CI)*	*p-value*	*HR (95% CI)*	*p-value*
1.00		0.91 (0.55 to 1.52)	0.720	0.79 (0.41 to 1.56)	0.504
1.00		2.37 (1.00 to 5.62)	0.051	1.73 (0.68 to 4.41)	0.254
1.00		1.93 (0.91 to 4.07)	0.086	1.65 (0.63 to 4.33)	0.305

*Variables include sex, dose used, CKD stage, history of GI bleed, ejection fraction, and RVSP

A multivariable Cox regression model was developed, controlling for current age, as well as a priori and confounding variables. With that, there was evidence of a 41% increase in bleeding with rivaroxaban compared to apixaban (HR 1.41 [1.04 to 1.90], *p* = 0.027) (Table 1A) and a statistically nonsignificant trend towards increased bleeding with dabigatran (HR 1.19 [0.82 to 1.72], *p* = 0.359).

A second model, incorporating the above variables and an interaction term for RVSP, was produced, and stratum-specific estimates were generated (Table 1B). There was a trend towards increased bleeding with increasing RVSP with people taking dabigatran and rivaroxaban, but not apixaban (Table 1C). In people with normal RVSP, there was no difference in bleeding rates across DOACs (Table 1D). In people with RVSP ≥ 45mmHg, bleeding rates were nearly twice as high in those taking rivaroxaban (HR 1.86 [1.09 to 3.19], *p* = 0.024) and dabigatran (HR 1.99 [1.02 to 3.88], *p* = 0.042). A third model with an interaction term for DOAC choice and LVEF was produced (Table 2). No statistically significant trends were noted. Incorporation of these interaction parameters did not significantly improve the fit of the association (RVSP test for interaction LR Chi^2^ [4] = 3.62, *p* = 0.459; LVEF test for interaction LR Chi^2^ [4] = 1.06, *p* = 0.900).

The proportion of bleeding events expected based on the ORBIT score was estimated for each RVSP threshold. There were 70.5%, 129% and 237% of predicted bleeds among people with RVSP < 35mmHg, 35 to 44mmHg, and ≥ 45mmHg, respectively [38].

#### Gastrointestinal bleeding

A similar modelling approach was used for gastrointestinal bleeding (SA Tables 2 and 4). The model produced included current age, sex, dose used, GI bleeding, RVSP category, CKD stage, and LVEF category. There were 153 events over 5,165.12 person-years, with a rate of 2.96 (2.53 to 3.47) gastrointestinal bleeds per 100 person-years (mean follow-up duration, 2.23 years).

**Table 4:** Hazard ratio for Gastrointestinal bleeding in people with NVAF receiving DOACs, stratified by DOAC choice, calculated using multivariable Cox regression controlling for current age and multiple confounders*. Stratum specific estimates are included in a subsequent model with additional parameters for interaction for EF and DOAC choice, calculated using linear computation

**A: Estimates without interaction term**					
Apixaban		Rivaroxaban		Dabigatran	
*HR (95% CI)*	*p-value*	*HR (95% CI)*	*p-value*	*HR (95% CI)*	*p-value*
1.00		1.32 (0.90 to 1.92)	0.157	1.12 (0.70 to 1.81)	0.637
**B: Estimates with interaction parameters for EF category and DOAC choice**					
Ejection Fraction					
1.00		1.22 (0.79 to 1.86)	0.369	1.02 (0.58 to 1.78)	0.945
1.47 (0.63 to 3.44)	0.379	1.61 (0.63 to 4.09)	0.318	0.77 (0.12 to 5.62)	0.795
1.15 (0.53 to 2.49)	0.730	2.85 (1.17 to 6.92)	0.021	2.47 (1.08 to 5.66)	0.032

*variables include sex, dose used, CKD stage, history of GI bleed, ejection fraction, and RVSP.

In the adjusted model, there was no significant difference between DOAC choice and GI bleeding rates (Table 3A). Incorporating interaction for DOAC choice and bleeding rates, there was substantial evidence of effect modification with increased bleeding rates with increased RVSP for dabigatran and rivaroxaban, but not apixaban (Table 3B). There was also strong evidence of interaction with ejection fraction: individuals with reduced LVEF who were on rivaroxaban or dabigatran had more than twice as many GI bleeds as those on apixaban (Table 4).

#### Intracranial hemorrhage

There were 18 bleeds over 5,440.81 person-years. Intracranial Hemorrhage incidence rate was 0.33 (0.21 to 0.53) per 100-person-years (mean follow-up duration 2.36 years). There was no evident pattern of bleeding rate difference across strata of DOAC and RVSP choice, as indicated by Mantel-Haenszel hazard ratios (Table 5).

**Table 5:** Hazard ratio for intracranial bleeding in people with NVAF receiving DOACs, stratified by DOAC choice, calculated using bivariable Cox regression controlling for current age. Stratum specific estimates are included in a subsequent model with additional parameters for interaction for RVSP category and DOAC choice, calculated using linear computation

**A: Estimates without interaction term**					
Apixaban		Apixaban		Apixaban	
*HR (95% CI)*	*p-value*	*HR (95% CI)*	*p-value*	*HR (95% CI)*	*p-value*
1.00		0.57 (0.15 to 2.15)	0.400	2.24 (0.81 to 6.18)	0.109
**B: Estimates with interaction parameters for RVSP and DOAC choice**					
RVSP					
1.00		0.95 (0.16 to 5.69)	0.955	3.77 (0.84 to 16.92)	0.083
1.26 (0.21 to 7.61)	0.803	1.24 (0.13 to 11.97)	0.852	1.73 (0.18 to 16.87)	0.637
2.96 (0.58 to 15.11)	0.192	**		5.36 (0.86 to 33.38)	0.072

** Unable to estimate due to data sparsity.

#### Stroke

There were 68 stroke incident events observed over 3,977.16 person-years (mean follow-up duration: 1.72 years). The stroke incidence rate was 1.71 (1.35 to 2.17) per 100 person-years. Variables associated with stroke rate included dose indication category, hyperlipidemia, and RVSP (SA Table 5). Variables that altered the association between DOAC choice and stroke rate included dose indication category, atrial flutter, paroxysmal atrial fibrillation, CKD stage, sex, COPD, RVSP, and weight category (Table 6).

**Table 6:** Hazard ratios for stroke rate among people with NVAF by DOAC choice, calculated using multivariable Cox regression controlling for current age and multiple confounders*. Stratum specific rates are included in a subsequent model with interaction for RVSP and DOAC choice using linear computation

**A: Estimates without interaction term**					
Apixaban		Rivaroxaban		Rivaroxaban	
*HR (95% CI)*	*p-value*	*HR (95% CI)*	*p-value*	*HR (95% CI)*	*p-value*
1.00		0.63 (0.34 to 1.18)	0.149	1.06 (0.53 to 2.09)	0.876
**B: With interaction parameters for RVSP and DOAC choice, compared to Apixaban with normal RVSP**					
Apixaban		Rivaroxaban		Rivaroxaban	
*HR (95% CI)*	*p-value*	*HR (95% CI)*	*p-value*	*HR (95% CI)*	*p-value*
1.00		0.65 (0.26 to 1.63)	0.363	1.11 (0.42 to 2.92)	0.827
1.98 (0.91 to 4.34)	0.086	2.18 (0.83 to 5.72)	0.114	1.46 (0.41 to 5.13)	0.559
2.54 (1.11 to 5.80)	0.027	0.58 (0.12 to 2.73)	0.491	4.40 (1.21 to 16.04)	0.025
**C: HR examining rate changes within each DOAC with increasing RVSP**					
Apixaban		Rivaroxaban		Rivaroxaban	
*HR (95% CI)*	*p-value*	*HR (95% CI)*	*p-value*	*HR (95% CI)*	*p-value*
1.00		1.00		1.00	
1.98 (0.91 to 4.34)	0.086	3.33 (1.12 to 9.88)	0.030	1.31 (0.32 to 5.31)	0.560
2.54 (1.11 to 5.80)	0.027	0.89 (0.17 to 4.56)	0.887	3.95 (0.94 to 16.72)	0.062
**D: HR examining rate changes across each stratum of increasing RVSP**					
Apixaban		Rivaroxaban		Rivaroxaban	
*HR (95% CI)*	*p-value*	*HR (95% CI)*	*p-value*	*HR (95% CI)*	*p-value*
1.00		0.65 (0.26 to 1.63)	0.363	1.11 (0.42 to 2.92)	0.827
1.00		1.10 (0.41 to 2.97)	0.854	0.73 (0.20 to 2.68)	0.639
1.00		0.23 (0.05 to 1.08)	0.063	1.73 (0.46 to 6.52)	0.416

*variables include sex, RVSP category, dose indicated, CKD stage, COPD, paroxysmal AF and atrial flutter

Controlling for confounders, there was a non-significant trend towards lower stroke rates in people on rivaroxaban (HR 0.63 [0.34 to 1.18], *p* = 0.149) compared to apixaban (Table 6A). There was no difference in stroke rates between dabigatran and apixaban (HR 1.06 [0.53 to 2.09], *p* = 0.876). When an interaction term for DOAC choice and RVSP was incorporated, there was an increase in the rate of stroke with RVSP ≥ 45mHg among people taking apixaban (HR 2.54 [1.11 to 5.80], *p* = 0.027) and dabigatran (HR 3.95 [0.94 to 16.72], *p* = 0.062), compared to people on the same anticoagulant with RVSP < 35mmHg (Table 6C). The trend among people taking rivaroxaban was not linear, and there was an increase in stroke rates among those with an RVSP 35-44mmHg compared to those with an RVSP < 35mmHg (HR 3.33 [1.12 to 9.88], *p* = 0.030) (Table 6C). Paradoxically, there was no difference in stroke rates among people taking rivaroxaban who had an RVSP ≥ 45 mmHg (HR 0.89 [0.17 to 4.56], *p* = 0.887). The addition of interaction terms for RVSP and stroke rate did not improve the overall fit of the model (LR Chi2 [4] = 5.08, *p* = 0.279).

The predicted stroke rate was estimated using the CHA_2_DS_2_-VASC score. Stroke rates were 28% predicted in people with RVSP < 35mmHg, 53.5% predicted among people with RVSP 35-44mmHg, and 54% among people with RVSP 45mmHg or greater [39].

## Discussion

This study verifies the excess bleeding in dabigatran and rivaroxaban relative to apixaban seen in prior studies [15, 20, 21]. The data presented also support the hypothesis that cardiac hemodynamics correlate with DOAC-specific outcomes. In the absence of elevated RVSP, there was no significant difference in outcomes between apixaban, dabigatran, and rivaroxaban. Among those with elevated RVSP, Apixaban was associated with significantly fewer bleeding events and more stroke events. In this cohort, rivaroxaban was associated with a higher incidence of bleeding events and a lower incidence of strokes. Dabigatran was associated with more bleeding and stroke events. A similar trend is observed in increased bleeding rates with dabigatran and rivaroxaban in patients with moderately reduced or reduced ejection fraction, and this persists when examining only gastrointestinal bleeds.

PH significantly impacts the performance of the CHA_2_DS_2_-VASC and ORBIT scores. As our cohort exclusively contains people on anticoagulation, it is expected that people would have a lower rate of stroke than predicted. Among people with low RVSP, the stroke rate is significantly lower, while it is greater than 50% predicted in the moderate and high RVSP groups. The predicted bleeding rate among people with high RVSP was more than twice that predicted by the ORBIT score.

While acknowledging the limitations of this observational, retrospective study, which do not allow for a determination of causation, we propose a biologically plausible explanation for this correlation that might account for the overall trend towards reduced bleeding with apixaban, as demonstrated in other studies.

### Mechanism of increased bleeding rates in heart failure

Bleeding and stroke rates are increased in heart failure [27]. There are several possible explanations for this. Impaired cardiac output could result in delivery of drugs to the liver and kidneys from systemic circulation [22]. Altered organ perfusion and hemodynamic parameters because of heart failure and pulmonary hypertension treatments may further result in bioaccumulation of medications including DOACs [22, 26, 40]. Finally, increased hepatic and renal congestion may result in increased rates of organ dysfunction over time. This may not be adequately captured using baseline laboratory investigations in cohort studies, resulting in residual confounding in time-series analysis [22, 41, 42].

### Mechanisms of differential absorption of DOACs

Drug absorption from the GI tract is determined by chemical properties of the drug. Apixaban is highly soluble, and its absorption is permeability limited (Biopharmaceutical (BPS) class III) [43, 44]. Dabigatran and rivaroxaban are highly permeable but with low solubility (BPS class II) so their absorption will be solubility limited [45, 46]. In congestive heart failure, permeability is reduced. Intestinal absorption of drugs may be directly impeded by intestinal edema [22]. In addition to edema, intestinal wall tissue in heart failure patients has a significantly greater collagen content, likely due to chronic mucosal hypoxia and hypoperfusion, which will further contribute to impaired permeability [47]. Additionally, hypoxia-mediated transcription of p-glycoprotein membrane carrier proteins in pulmonary hypertension and heart failure reduces permeability through increased intestinal drug excretion [48–50]. We hypothesize that apixaban absorption is reduced in heart failure and pulmonary hypertension, resulting in lower rates of bleeding.

Our results show that stroke rates are higher in people with elevated RVSP taking apixaban and dabigatran compared to those taking rivaroxaban. That apixaban is associated with more stroke events supports the hypothesis that impaired absorption accounts for lower bleeding rates in heart failure. Excess bleeding and stroke rates in people with raised RVSP taking dabigatran could be explained by greater temporal fluctuations in dabigatran levels with increased caval pressures due to very high renal excretion (80%) relative to apixaban (25%) and rivaroxaban (36%), in addition to more variable expression of itssactivating enzyme, carboxylesterase 1, in pulmonary hypertension [51–53].

### Strengths

This study supports an association between dabigatran and rivaroxaban use in right heart failure and major bleeding events. Objective exposure measures such as TTE measurements, and standardized diagnostic criteria for major bleeding events were applied. The results were hypothesis driven. The trend towards increased bleeding with increasing RVSP in people using dabigatran and rivaroxaban showed a clear biologic gradient. Through use of a cohort design with routine data documented prior to the event, there is evidence of temporality for DOAC initiation with increased bleeding rates in these subgroups. Use of individualized multivariable regression approaches for each outcome further reduces the risk of confounding.

### Study strengths

This study has several strengths, including pragmatic design, relying on routine data that would typically be available at the point of first anticoagulant decision making and observing real world outcomes. This study is unique in presenting clinical data accompanied by detailed echocardiographic measurements, which are not routinely available in post-marketing surveillance datasets, while retaining a large sample size. Use of multiple RVSP strata allows assessment of a biologic gradient and thus enables better appraisal of associations.

### Study limitations

**Fig. 2:**
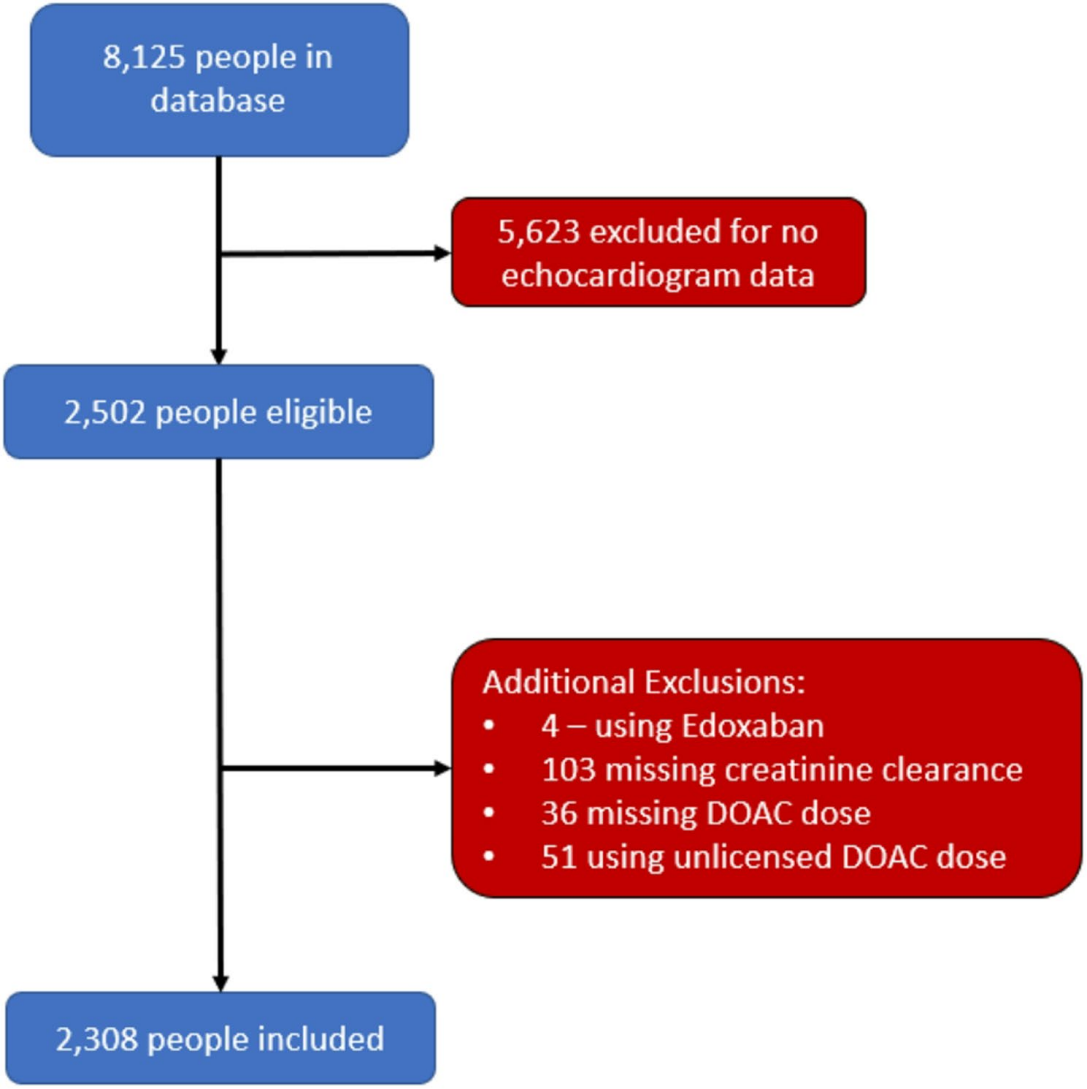
Graphical abstract. Bleeding hazard ratios (HR) in a cohort of people with nonvalvular atrial fibrillation taking different DOACs, with subsequent stratification by absence (right ventricular systolic pressure (RVSP) < 35mmHg) and presence (RVSP ≥ 45mmHg) of pulmonary hypertension

Acute or chronic illnesses not accounted for by this study design may have confounded the association between cardiac hemodynamics and DOAC-specific outcomes. Sampling bias is possible in this study as it derives results from routinely available hospital data. For example, the need for at least one follow-up record may exclude patients who do not experience an outcome, as they are less likely to return to the hospital, resulting in an overestimation of the true event rate. This study included any available echocardiogram completed at our institution, including those done not as part of initial evaluation of AF and those done for evaluation of another presentation, however a substantial number of patients were excluded due to lack of routine echo data, and this could impact accuracy of results (Fig. 2). Similarly, patient characteristics may have changed during the study, such as changes in creatinine clearance or progression from paroxysmal to persistent atrial fibrillation, and this may result in underestimation of effect sizes seen with risk factors for specific events.

One limitation of this study is the use of non-invasive hemodynamic measurements to estimate true RVSP. Use of routinely collected right heart catheterization data would likely result in significant sampling bias as it is not routinely collected during atrial fibrillation evaluation. Transthoracic echocardiogram is more frequently performed in evaluation of atrial fibrillation, so it is less likely to produce selection bias. While RVSP estimates from TTE can be unreliable with minimal RVSP or with torrential TR, at mid-range pressures such as the thresholds in this study, echocardiographic measurements are strongly correlated with invasive hemodynamic measurements [54–58].

## Conclusion

This study demonstrates that, in people with NVAF with elevated RVSP on echocardiogram, there is a correlation between use of apixaban and increased stroke and fewer bleeding events compared to rivaroxaban, with the caveat of higher thromboembolic risk. Equally people taking rivaroxaban with elevated RVSP may have higher rates of major bleeding but with lower risk of stroke. Anticoagulation selection in this circumstance should rely on individual risk-assessment and informed shared-decision making. Among people taking dabigatran, there is a relatively higher rate of bleeding and stroke compared to apixaban in this cohort. In the absence of elevated RVSP and normal LVEF, there is no significant difference in DOAC performance with respect to clinical outcomes. Existing knowledge about alterations in drug pharmacokinetics provides a biologically plausible explanation for this correlation, and there are many analogous instances of altered bioavailability in heart failure and PH.

## Supplementary Information

Supplementary Material 1.

## Data Availability

The data underlying this article will be shared on a reasonable request to the corresponding author.

## Abbreviations

AF: Atrial fibrillation or atrial flutter
NVAF: Non–valvular atrial fibrillation or atrial flutter
DOAC: Direct oral anticoagulant
RVSP: Right ventricular systolic pressure
LVEF: Left Ventricular Ejection Fraction
PH: Pulmonary hypertension
GI: Gastrointestinal
DM: Diabetes mellitus
TIA: Transient Ischemic attack
CKD: Chronic kidney disease
