# Supplementary material for: Echocardiographic hemodynamics correlate with differences in DOAC-specific bleeding and stroke rates in non-valvular atrial fibrillation
Source: BMC Cardiovasc Disord. 2025 Oct 28;25:774. doi: 10.1186/s12872-025-05239-w (PMC12570840; doi:10.1186/s12872-025-05239-w)
Supplement: Supplementary file 1 — Supplementary Material 1. [file 12872_2025_5239_MOESM1_ESM.docx]

**SUPPLEMENTAL APPENDIX**

| Table 1: Association between DOAC choice and clinical variables | | | | | | |
| --- | --- | --- | --- | --- | --- | --- |
|  |  | **Apixaban** | **Rivaroxaban** | **Dabigatran** | **Test for heterogeneity** | |
|  |  | **N (%)** | **N (%)** | **N (%)** | **Chi^2^ (d.f)** | **p-value** |
| Sex | |  |  |  | 8.972 (2) | 0.011 |
|  | Male | 657 (50.46%) | 370 (53.39%) | 187 (59.74%) |  |  |
|  | Female | 645 (49.54%) | 323 (46.61%) | 126 (40.26%) |  |  |
| Age at entry | |  |  |  | 31.847 (8) | <0.001 |
|  | 0 to 64 | 215 (16.51%) | 157 (22.66%) | 68 (21.73%) |  |  |
|  | 65 to 69 | 209 (16.05%) | 131 (18.9%) | 52 (16.61% |  |  |
|  | 70 to 74 | 250 (19.20%) | 144 (20.78%) | 58 (18.53%) |  |  |
|  | 75 to 79 | 254 (19.51%) | 117 (16.88%) | 71 (22.68%) |  |  |
|  | 80 + | 374 (28.73%) | 144 (20.78%) | 64 (20.45%) |  |  |
| Dose used | |  |  |  | 16.160 (2) | <0.001 |
|  | Normal | 974 (74.81%) | 566 (81.67%) | 257 (82.11%) |  |  |
|  | Reduced | 328 (25.19%) | 127 (18.33%) | 56 (17.89%) |  |  |
| Atherosclerotic disease | | |  |  | 0.339 (2) | 0.339 |
|  | No | 1,153 (88.56%) | 624 (90.04%) | 285 (91.05%) |  |  |
|  | Yes | 149 (11.44%) | 69 (9.96%) | 28 (8.95%) |  |  |
| Atrial flutter | |  |  |  | 13.177 (2) | 0.001 |
|  | Absent | 1,100 (84.49%) | 544 (78.50%) | 247 (78.91%) |  |  |
|  | Present | 202 (15.51%) | 149 (21.50%) | 66 (21.09%) |  |  |
| Paroxysmal atrial fibrillation | | |  |  | 68.362 (2) | <0.001 |
|  | Absent | 370 (28.42%) | 131 (18.9%) | 25 (7.99%) |  |  |
|  | Present | 932 (71.58%) | 562 (81.10%) | 288 (92.01%) |  |  |
| Creatinine clearance (eGFR) | | |  |  | 43.613 (8) | <0.001 |
|  | >90 | 165 (12.67%) | 130 (18.76%) | 51 (16.29%) |  |  |
|  | 60 to 90 | 441 (33.87%) | 269 (38.82%) | 139 (44.41%) |  |  |
|  | 45 to 60 | 342 (26.27%) | 154 (22.22%) | 72 (23.00%) |  |  |
|  | 30 to 45 | 260 (19.97%) | 107 (15.44%) | 43 (13.74%) |  |  |
|  | <30 | 94 (7.22%) | 33 (4.67%) | 8 (2.56%) |  |  |
| COPD | | |  |  | 2.644 (2) | 0.267 |
|  | No | 996 (76.5%) | 509 (73.45%) | 231 (73.80%) |  |  |
|  | Yes | 306 (23.5%) | 184 (26.55%) | 82 (26.2%) |  |  |
| Hypertension | | |  |  | 0.206 (2) | 0.902 |
|  | Absent | 179 (13.75%) | 98 (14.14%) | 46 (14.70%) |  |  |
|  | Present | 1,123 (86.25%) | 595 (85.86%) | 267 (85.30%) |  |  |
| Hyperlipidemia | |  |  |  | 0.972 (2) | 0.615 |
|  | Absent | 384 (29.49%) | 196 (28.28%) | 98 (31.31%) |  |  |
|  | Present | 918 (70.51%) | 497 (71.72%) | 215 (68.69%) |  |  |
| Diabetes mellitus | | |  |  | 1.555 (2) | 0.46 |
|  | Absent | 952 (73.12%) | 491 (70.85%) | 221 (70.61%) |  |  |
|  | Present | 350 (26.88%) | 202 (29.15%) | 92 (29.39%) |  |  |
| History of hemorrhagic stroke | | |  |  | 2.073 (2) | 0.355 |
|  | No | 1,281 (98.39%) | 685 (98.85%) | 311 (99.36%) |  |  |
|  | Yes | 21 (1.61%) | 8 (1.15%) | 2 (0.64%) |  |  |
| History of GI bleeding | | |  |  | 6.61 (2) | 0.037 |
|  | No | 1,171 (89.94%) | 637 (91.92%) | 289 (92.33%) |  |  |
|  | Yes | 131 (10.06%) | 56 (8.08%) | 21 (9.14%) |  |  |
| Prior ischemic stroke/TIA | | |  |  | 10.584 (4) | 0.032 |
|  | None | 916 (70.35%) | 527 (76.05%) | 231 (73.80%) |  |  |
|  | TIA | 54 (4.15%) | 33 (4.76%) | 14 (4.47%) |  |  |
|  | Stroke | 332 (25.50%) | 133 (19.19%) | 68(21.73%) |  |  |
| Race | |  |  |  | 3.573 (2) | 0.168 |
|  | White | 1,231 (94.55%) | 664 (95.82%) | 303 (96.81%) |  |  |
|  | Non-white | 71 (5.45%) | 29 (4.18%) | 10 (3.19%) |  |  |
| Weight category | | |  |  | 17.135 (4) | 0.002 |
|  | <60kg | 154 (11.83%) | 56 (8.08%) | 20 (6.39%) |  |  |
|  | 60 to 120kg | 1,052 (80.80%) | 564 (81.39%) | 262 (83.71%) |  |  |
|  | >120kg | 96 (7.37%) | 73 (10.53%) | 31 (9.90%) |  |  |
| Prosthetic valve | | |  |  | 0.007 (2) | 0.997 |
|  | No | 1,255 (96.39%) | 668 (96.39%) | 302 (96.49%) |  |  |
|  | Yes | 47 (3.61%) | 25 (3.61%) | 11 (3.51%) |  |  |
| RVSP category | |  |  |  | 8.610 (4) | 0.072 |
|  | 0 to 34 | 661 (50.77%) | 392 (56.57%) | 152 (48.56%) |  |  |
|  | 35 to 44 | 348 (26.73%) | 159 (22.94%) | 91 (29.07%) |  |  |
|  | 45 + | 293 (22.50%) | 142 (20.49%) | 70 (22.36%) |  |  |
| EF category | |  |  |  | 9.232 (4) | 0.056 |
|  | >=50% | 1,012 (77.73%) | 548 (79.08%) | 248 (79.23%) |  |  |
|  | 41 to 49% | 110 (8.45%) | 76 (10.97%) | 25 (7.99%) |  |  |
|  | <=40% | 180 (13.82%) | 69 (9.96%) | 40 (12.78%) |  |  |

| **Table 2: Association between clinical variables and major bleeding, with hazard ratios and Chi^2^ test for heterogeneity, for multiple variables.** | | | | | | | |
| --- | --- | --- | --- | --- | --- | --- | --- |
|  |  | **MAJOR BLEEDING** | | | **GASTROINTESTINAL BLEEDING** | | |
|  |  | **Rate** | **Hazard Ratio** | **Chi^2^ test** | **Rate** | **Hazard Ratio** | **Chi^2^ test** |
|  |  | **(per 100 years)** | **HR (95% CI)** | **p-value** | **(per 100 years)** | **HR (95% CI)** | **p-value** |
| DOAC name | |  |  |  |  |  |  |
|  | Apixaban | 2.75 (2.30 to 3.29) | 1.00 |  | 2.79 (2.20 to 3.54) | 1.00 |  |
|  | Rivaroxaban | 3.36 (2.73 to 4.14) | 1.22 (0.93 to 1.61) | 0.155 | 3.01 (2.26 to 4.01) | 1.08 (0.74 to 1.56) | 0.690 |
|  | Dabigatran | 2.83 (2.06 to 3.87) | 1.03 (0.72 to 1.47) | 0.890 | 2.74 (1.85 to 4.05) | 0.98 (0.62 to 1.55) | 0.933 |
| Age category | |  |  |  |  |  |  |
|  | 0 to 64 | 2.46 (1.74 to 3.48) | 1.00 |  | 1.87 (1.09 to 3.23) | 1.00 |  |
|  | 65 to 69 | 1.18 (0.70 to 2.00) | 0.48 (0.26 to 0.90) | 0.019 | 1.02 (0.49 to 2.14) | 0.55 (0.22 to 1.37) | 0.189 |
|  | 70 to 74 | 2.71 (2.03 to 3.62) | 1.10 (0.70 to 1.73) | 0.670 | 2.92 (2.04 to 4.18) | 1.56 (0.81 to 2.99) | 0.177 |
|  | 75 to 79 | 2.78 (2.09 to 3.70) | 1.13 (0.72 to 1.77) | 0.589 | 3.50 (2.51 to 4.87) | 1.87 (0.99 to 3.53) | 0.051 |
|  | 80+ | 4.31 (3.58 to 5.21) | 1.75 (1.18 to 2.60) | 0.005 | 3.87 (3.05 to 4.90) | 2.06 (1.14 to 3.73) | 0.015 |
| Sex | |  |  |  |  |  |  |
|  | Male | 2.73 (2.30 to 3.25) | 1.00 |  | 2.71 (2.15 to 3.42) | 1.00 |  |
|  | Female | 3.24 (2.71 to 3.88) | 1.19 (0.92 to 1.52) | 0.181 | 3.01 (2.38 to 3.81) | 1.11 (0.797 to 1.542) | 0.540 |
| Dose used | |  |  |  |  |  |  |
|  | Normal | 2.77 (2.40 to 3.20) | 1.00 |  | 2.57 (2.12 to 3.11) | 1.0 |  |
|  | Adjusted | 3.67 (2.87 to 4.70) | 1.32 (1.00 to 1.76) | 0.053 | 4.15 (3.01 to 5.73) | 1.62 (1.11 to 2.35) | 0.011 |
| Dose indicated | |  |  |  |  |  |  |
|  | Normal | 2.68 (2.33 to 3.09) | 1.00 |  | 2.70 (2.25 to 3.24) | 1.00 |  |
|  | Adjusted | 3.96 (2.87 to 5.46) | 1.48 (1.04 to 2.10) | 0.029 | 3.70 (2.36 to 5.80) | 1.37 (0.84 to 2.22) | 0.202 |
|  | Contraindicated | 6.66 (4.19 to 10.57) | 2.48 (1.53 to 4.02) | <0.001 | 4.29 (1.93 to 9.56) | 1.59 (0.70 to 3.61) | 0.265 |
| Atherosclerotic disease | | |  |  |  |  |  |
|  | Absent | 2.94 (2.58 to 3.37) | 1.00 |  | 2.75 (2.30 to 3.29) | 1.00 |  |
|  | Present | 3.01 (2.12 to 4.28) | 1.02 (0.70 to 1.49) | 0.91 | 3.72 (2.37 to 5.83) | 1.35 (0.83 to 2.19) | 0.221 |
| Atrial flutter | |  |  |  |  |  |  |
|  | Absent | 3.06 (2.65 to 3.52) | 1.00 |  | 2.83 (2.35 to 3.41) | 1.00 |  |
|  | Present | 2.67 (2.07 to 3.45) | 0.87 (0.65 to 1.17) | 0.367 | 2.93 (2.06 to 4.16) | 1.03 (0.69 to 1.54) | 0.872 |
| Paroxysmal atrial fibrillation | | |  |  |  |  |  |
|  | Absent | 3.29 (2.34 to 4.62) | 1.00 |  | 2.44 (1.59 to 3.74) | 1.00 |  |
|  | Present | 2.91 (2.55 to 3.33) | 0.89 (0.51 to 1.28) | 0.515 | 2.94 (2.46 to 3.51) | 1.21 (0.76 to 1.92) | 0.430 |
| Creatinine clearance | | |  |  |  |  |  |
|  | >90 | 2.24 (1.55 to 3.24) | 1.00 |  | 1.66 (0.98 to 2.80) | 1.00 |  |
|  | 60 to 90 | 2.42 (1.94 to 3.00) | 1.08 (0.70 to 1.66) | 0.730 | 2.30 (1.73 to 3.06) | 1.39 (0.77 to 2.52) | 0.279 |
|  | 45 to 60 | 3.24 (2.54 to 4.13) | 1.45 (0.93 to 2.25) | 0.10 | 3.62 (2.67 to 4.92) | 2.19 (1.19 to 4.01) | <0.001 |
|  | 30 to 45 | 3.64 (2.76 to 4.80) | 1.62 (1.02 to 2.58) | 0.038 | 3.99 (2.78 to 5.75) | 2.41 (1.27 to 4.56) | 0.005 |
|  | <30 | 4.09 (3.93 to 8.74) | 2.62 (1.52 to 4.51) | <0.001 | 5.01 (2.70 to 9.31) | 3.025 (1.34 to 6.81) | 0.005 |
| COPD | |  |  |  |  |  |  |
|  | Absent | 2.75 (2.37 to 3.20) | 1.00 |  | 2.59 (2.13 to 3.16) |  |  |
|  | Present | 3.53 (2.82 to 4.40) | 1.28 (0.98 to 1.68) | 0.069 | 3.69 (2.74 to 4.98) | 1.43 (1.00 to 2.04) | 0.052 |
| Hypertension | |  |  |  |  |  |  |
|  | Absent | 2.30 (1.50 to 3.52) | 1.00 |  | 2.00 (1.16 to 3.45) | 1.00 |  |
|  | Present | 3.03 (2.66 to 3.46) | 1.32 (0.85 to 2.07) | 0.220 | 2.98 (2.51 to 3.54) | 1.49 (0.84 to 2.63) | 0.169 |
| Hyperlipidemia | |  |  |  |  |  |  |
|  | Absent | 3.36 (2.60 to 4.33) | 1.00 |  | 2.99 (2.17 to 4.13) | 1.00 |  |
|  | Present | 2.85 (2.47 to 3.28) | 0.85 (0.63 to 1.14) | 0.268 | 2.80 (2.31 to 3.40) | 0.94 (0.64 to 1.36) | 0.732 |
| Diabetes mellitus | |  |  |  |  |  |  |
|  | Absent | 2.79 (2.39 to 3.25) | 1.00 |  | 2.78 (2.28 to 3.38) | 1.00 |  |
|  | Present | 3.35 (2.70 to 4.16) | 1.20 (0.92 to 1.57) | 0.170 | 3.05 (2.24 to 4.14) | 1.10 (0.76 to 1.58) | 0.618 |
| History of GI bleed | |  |  |  |  |  |  |
|  | Absent | 2.61 (2.26 to 3.01) | 1.00 |  | 2.25 (1.86 to 2.74) | 1.00 |  |
|  | Present | 5.21 (4.03 to 6.74) | 2.00 (1.49 to 2.68) | <0.001 | 8.60 (6.31 to 11.72) | 3.81 (2.64 to 5.50) | <0.001 |
| Prior ischemic stroke/TIA | | |  |  |  |  |  |
|  | No | 2.82 (2.43 to 3.28) | 1.00 |  | 2.49 (2.04 to 3.05) | 1.00 |  |
|  | TIA | 3.09 (1.75 to 5.44) | 1.10 (0.61 to 1.97) | 0.761 | 4.38 (2.28 to 8.42) | 1.76 (0.89 to 3.48) | 0.102 |
|  | Stroke | 3.35 (2.62 to 4.27) | 1.19 (0.89 to 1.58) | 0.241 | 3.92 (2.85 to 5.39) | 1.57 (1.08 to 2.29) | 0.018 |
| Race | |  |  |  |  |  |  |
|  | White | 2.92 (2.57 to 3.32) | 1.00 |  | 2.85 (2.41 to 3.37) | 1.00 |  |
|  | Non-white | 4.08 (2.20 to 7.59) | 1.40 (0.74 to 2.63) | 0.298 | 2.86 (1.19 to 6.86) | 1.00 (0.41 to 2.45) | 0.997 |
| Weight category | |  |  |  |  |  |  |
|  | <60kg | 6.81 (2.36 to 5.26) | 1.22 (0.80 to 1.87) | 0.354 | 3.20 (1.82 to 5.63) | 1.14 (0.63 to 2.07) | 0.664 |
|  | 60 to 120kg | 2.88 (2.51 to 3.31) | 1.00 |  | 2.80 (2.34 to 3.36) | 1.00 |  |
|  | >120kg | 3.08 (2.06 to 4.59) | 1.07 (0.70 to 1.63) | 0.765 | 3.00 (1.74 to 5.17) | 1.07 (0.60 to 1.90) | 0.817 |
| Prosthetic valve | |  |  |  |  |  |  |
|  | Absent | 2.91 (2.56 to 3.31) | 1.00 |  | 2.78 (2.34 to 3.29) | 1.00 |  |
|  | Present | 3.90 (2.35 to 6.47) | 1.34 (0.80 to 2.26) | 0.269 | 4.77 (2.48 to 9.17) | 1.72 (0.88 to 3.38) | 0.112 |
| RVSP | |  |  |  |  |  |  |
|  | 0 to 34 | 2.40 (2.00 to 2.87) | 1.00 |  | 2.51 (2.00 to 3.14) | 1.00 |  |
|  | 35 to 44 | 3.03 (2.34 to 3.92) | 1.26 (0.92 to 1.73) | 0.144 | 2.47 (1.72 to 3.56) | 0.99 (0.64 to 1.51) | 0.945 |
|  | 45+ | 4.62 (3.67 to 5.82) | 1.93 (1.44 to 2.58) | <0.001 | 4.85 (3.50 to 6.73) | 1.93 (1.30 to 2.88) | <0.001 |
| EF category | |  |  |  |  |  |  |
|  | >=50% | 2.86 (2.48 to 3.29) | 1.00 |  | 2.66 (2.20 to 3.21) | 1.00 |  |
|  | 41 to 49% | 2.96 (1.93 to 4.55) | 1.04 (0.66 to 1.63) | 0.874 | 2.96 (1.68 to 5.21) | 1.11 (0.61 to 2.02) | 0.724 |
|  | <=40% | 3.71 (2.63 to 5.25) | 1.30 (0.90 to 1.89) | 0.168 | 4.38 (2.86 TO 6.72) | 1.65 (1.03 to 2.63) | 0.034 |

| Table 3: Cox bivariable regression for the association between anticoagulant choice and rate of first bleeding event, controlling for current age and each variable in turn among people being treated with a DOAC for stroke prevention in NVAF. | | | | | | |
| --- | --- | --- | --- | --- | --- | --- |
|  |  | **Apixaban** | **Rivaroxaban** |  | **Dabigatran** |  |
|  |  | ***HR*** | ***HR (95% CI)*** | ***p-value*** | ***HR (95% CI)*** | ***p-value*** |
| Unadjusted | | 1.00 | 1.30 (0.99 to 1.72) | 0.060 | 1.09 (0.76 to 1.57) | 0.632 |
| Sex | | 1.00 | 1.31 (0.99 to 1.73) | 0.058 | 1.11 0.77 to 1.61) | 0.564 |
| Dose used | | 1.00 | 1.30 (0.99 to 1.72) | 0.062 | 1.09 (0.76 to 1.57) | 0.632 |
| Dose indicated | | 1.00 | 1.27 (0.96 to 1.70) | 0.096 | 1.11 (0.77 to 1.60) | 0.576 |
| CKD category | | 1.00 | 1.33 (1.00 to 1.75) | 0.048 | 1.13 (0.78 to 1.63) | 0.521 |
| COPD | | 1.00 | 1.29 (0.98 to 1.71) | 0.068 | 1.09 (0.76 to 1.58) | 0.627 |
| GI bleed | | 1.00 | 1.38 (1.04 to 1.83) | 0.024 | 1.13 (0.79 to 1.63) | 0.506 |
| Prior stroke/TIA | | 1.00 | 1.31 (0.99 to 1.73) | 0.057 | 1.10 (0.76 to 1.58) | 0.620 |
| Weight category | | 1.00 | 1.30 (0.98 to 1.71) | 0.066 | 1.09 (0.76 to 1.57) | 0.645 |
| Prosthetic valve | | 1.00 | 1.30 (0.99 to 1.72) | 0.061 | 1.09 (0.76 to 1.57) | 0.644 |
| RVSP | | 1.00 | 1.30 (0.99 to 1.72) | 0.063 | 1.09 (0.76 to 1.57) | 0.633 |
| EF | | 1.00 | 1.31 (0.99 to 1.73) | 0.055 | 1.08 (0.75 to 1.56) | 0.664 |
| Atherosclerosis | | 1.00 | 1.30 (0.99 to 1.72) | 0.061 | 1.09 (0.76 to 1.57) | 0.639 |
| HTN | | 1.00 | 1.31 (0.99 to 1.73) | 0.058 | 1.09 (0.76 to 1.57) | 0.058 |
| HLD | | 1.00 | 1.31 (0.99 to 1.73) | 0.055 | 1.09 (0.76 to 1.57) | 0.641 |
| Atrial flutter | | 1.00 | 1.31 (0.99 to 1.72) | 0.060 | 1.10 (0.76 to 1.58) | 0.615 |
| Paroxysmal AF | | 1.00 | 1.31 (1.00 to 1.73) | 0.059 | 1.10 (0.76 to 1.59) | 0.614 |
| DM | | 1.00 | 1.31 (0.99 to 1.73) | 0.059 | 1.10 (0.76 to 1.58) | 0.61 |
| Hemorrhagic stroke | | 1.00 | 1.30 (0.99 to 1.72) | 0.063 | 1.08 (0.75 to 1.55) | 0.683 |
| Nonwhite race | | 1.00 | 1.31 (0.99 to 1.73) | 0.055 | 1.10 (0.76 to 1.58) | 0.608 |

| **Table 4: Cox bivariable regression controlling for current age and each variable in turn for association between DOAC choice and gastrointestinal bleeding rate among people taking DOACs for stroke prevention in NVAF.** | | | | | | |
| --- | --- | --- | --- | --- | --- | --- |
|  |  | **Apixaban** | **Rivaroxaban** |  | **Dabigatran** |  |
|  |  | ***HR*** | ***HR (95% CI)*** | ***p-value*** | ***HR (95% CI)*** | ***p-value*** |
|  |  |  |  |  |  |  |
| Unadjusted | | 1.00 | 1.15 (0.79 to 1.68) | 0.453 | 1.01 (0.63 to 1.60) | 0.980 |
| Sex | | 1.00 | 1.16 (0.79 to 1.68) | 0.449 | 1.01 (0.63 to 1.61) | 0.959 |
| Dose used | | 1.00 | 1.17 (0.80 to 1.70) | 0.413 | 1.00 (0.63 to 1.59) | 0.995 |
| Dose indicated | | 1.00 | 1.16 (0.79 to 1.70) | 0.462 | 1.01 (0.63 to 1.60) | 0.981 |
| Creatinine clearance | | 1.00 | 1.19 (0.82 to 1.73) | 0.365 | 1.08 (0.67 to 1.72) | 0.759 |
| COPD | | 1.00 | 1.15 (0.79 to 1.67) | 0.472 | 1.01 (0.64 to 1.61) | 0.959 |
| GI bleed | | 1.00 | 1.24 (0.85 to 1.80) | 0.262 | 1.03 (0.65 to 1.63) | 0.911 |
| Prior stroke/TIA | | 1.00 | 1.17 (0.81 to 1.71) | 0.402 | 1.02 (0.64 to 1.62) | 0.937 |
| Weight category | | 1.00 | 1.15 (0.79 to 1.68) | 0.455 | 1.01 (0.63 to 1.61) | 0.972 |
| Prosthetic valve | | 1.00 | 1.14 (0.78 to 1.66) | 0.491 | 0.99 (0.63 to 1.58) | 0.982 |
| RVSP | | 1.00 | 1.15 (0.79 to 1.68) | 0.461 | 1.04 (0.65 to 1.68) | 0.868 |
| EF | | 1.00 | 1.19 (0.81 to 1.73) | 0.376 | 0.99 (0.62 to 1.58) | 0.966 |
| Atherosclerosis | | 1.00 | 1.16 (0.80 to 1.69) | 0.432 | 1.02 (0.64 to 1.62) | 0.933 |
| HTN | | 1.00 | 1.16 (0.79 to 1.68) | 0.448 | 1.01 (0.63 to 1.60) | 0.979 |
| HLD | | 1.00 | 1.16 (0.80 to 1.69) | 0.434 | 1.01 (0.63 to 1.60) | 0.978 |
| Atrial flutter | | 1.00 | 1.15 (0.79 to 1.67) | 0.465 | 1.00 (0.63 to 1.59) | 0.993 |
| Paroxysmal afib | | 1.00 | 1.13 (0.78 to 1.65) | 0.518 | 0.96 (0.60 to 1.54) | 0.872 |
| DM | | 1.00 | 1.15 (0.79 to 1.68) | 0.452 | 1.01 (0.63 to 1.60) | 0.971 |
| Non-white race | | 1.00 | 1.15 (0.79 to 1.68) | 0.455 | 1.01 (0.63 to 1.60) | 0.973 |

| **Table 5: Association between clinical variables and stroke rate in a cohort of people taking DOACs for stroke prevention in NVAF.** | | | | |
| --- | --- | --- | --- | --- |
|  |  | **Rate** | **Hazard Ratio** | **Chi^2^ test** |
|  |  | **(per 100 person years)** | **HR (95% CI)** | **p-value** |
| DOAC name | |  |  |  |
|  | Apixaban | 2.01 (1.47 to 2.75) | 1.00 |  |
|  | Rivaroxaban | 1.24 (0.76 to 2.02) | 0.61 (0.34 to 1.10) | 0.097 |
|  | Dabigatran | 1.74 (1.01 to 3.01) | 0.87 (0.463 to 1.63) | 0.658 |
| Age category | |  |  |  |
|  | 0 to 64 | 1.27 (0.63 to 2.53) | 1.00 |  |
|  | 65 to 69 | 1.71 (0.92 to 3.17) | 1.35 (0.53 to 3.41) | 0.528 |
|  | 70 to 74 | 1.00 (0.52 to 1.92) | 0.79 (0.30 to 2.04) | 0.624 |
|  | 75 to 79 | 1.26 (0.70 to 2.27) | 0.99 (0.40 to 2.47) | 0.990 |
|  | 80+ | 2.15 (1.50 to 3.07) | 1.70 (0.78 to 3.70) | 0.179 |
| Sex | |  |  |  |
|  | Male | 1.47 (1.04 to 2.10) | 1.00 |  |
|  | Female | 1.97 (1.43 to 2.73) | 1.34 (0.83 to 2.16) | 0.228 |
| Dose used | |  |  |  |
|  | Normal | 1.57 (1.20 to 2.06) | 1.00 |  |
|  | Adjusted | 2.41 (1.48 to 3.94) | 1.54 (0.88 to 2.69) | 0.130 |
| Dose indicated | |  |  |  |
|  | Normal | 1.61 (1.24 to 2.08) | 1.00 |  |
|  | Adjusted | 1.76 (0.84 to 3.70) | 1.10 (0.50 to 2.41) | 0.817 |
|  | Contraindicated | 5.45 (2.27 to 13.09) | 3.39 (1.36 to 8.47) | 0.005 |
| Atherosclerotic disease | |  |  |  |
|  | Absent | 1.70 (1.33 to 2.18) | 1.00 |  |
|  | Present | 1.81 (0.81 to 4.03) | 1.06 (0.46 to 2.46) | 0.884 |
| Atrial flutter | |  |  |  |
|  | Absent | 1.88 (1.46 to 2.43) | 1.00 |  |
|  | Present | 1.07 (0.55 to 2.05) | 0.57 (0.28 to 1.14) | 0.107 |
| Paroxysmal atrial fibrillation | | |  |  |
|  | Absent | 2.41 (1.50 to 3.87) | 1.00 |  |
|  | Present | 1.56 (1.19 to 2.05) | 0.65 (0.37 to 1.12) | 0.118 |
| Creatinine clearance | |  |  |  |
|  | >90 | 1.64 (0.93 to 2.89) | 1.00 |  |
|  | 60 to 90 | 1.13 (0.72 to 1.78) | 0.69 (0.34 to 1.42) | 0.312 |
|  | 45 to 60 | 2.33 (1.52 to 3.58) | 1.42 (0.70 to 2.88) | 0.331 |
|  | 30 to 45 | 2.25 (1.28 to 3.96) | 1.37 (0.62 to 3.05) | 0.439 |
|  | <30 | 2.90 (1.09 to 7.73) | 1.77 (0.57 to 5.47) | 0.319 |
| COPD | |  |  |  |
|  | Absent | 1.90 (1.47 to 2.45) | 1.00 |  |
|  | Present | 1.03 (0.54 to 1.99) | 0.55 (0.27 to 1.10) | 0.085 |
| Hypertension | |  |  |  |
|  | Absent | 1.21(0.58 to 2.54) | 1.00 |  |
|  | Present | 1.79 (1.40 to 2.31) | 1.48 (0.68 to 3.24) | 0.321 |
| Hyperlipidemia | |  |  |  |
|  | Absent | 0.90 (0.48 to 1.67) | 1.00 |  |
|  | Present | 2.03 (1.57 to 2.62) | 2.26 (1.15 to 4.41) | 0.015 |
| Diabetes mellitus | |  |  |  |
|  | Absent | 1.94 (1.49 to 2.51) | 1.00 |  |
|  | Present | 1.06 (0.59 to 1.92) | 0.55 (0.29 to 1.05) | 0.065 |
| History of GI bleed | |  |  |  |
|  | Absent | 1.79 (1.40 to 2.28) | 1.00 |  |
|  | Present | 0.87 (0.28 to 2.68) | 0.48 (0.15 to 1.54) | 0.209 |
| Race | |  |  |  |
|  | White | 1.72 (1.35 to 2.19) |  |  |
|  | Non-white | 1.43 (0.36 to 5.72) | 0.83 (0.20 to 3.40) | 0.798 |
| Weight category | |  |  |  |
|  | <60kg | 3.25 (1.69 to 6.24) | 1.96 (0.97 to 3.96) | 0.057 |
|  | 60 to 120kg | 1.66 (1.28 to 2.16) | 1.00 |  |
|  | >120kg | 1.03 (0.39 to 2.74) | 0.62 (0.22 to 1.71) | 0.350 |
| Prosthetic valve | |  |  |  |
|  | Absent | 1.69 (1.33 to 2.15) | 1.00 |  |
|  | Present | 2.30 (0.74 to 7.14) | 1.36 (0.43 to 4.34) | 0.598 |
| RVSP | |  |  |  |
|  | 0 to 34 | 1.11 (0.77 to 1.61) | 1.00 |  |
|  | 35 to 44 | 2.46 (1.64 to 3.70) | 2.21 (1.27 to 3.83) | 0.004 |
|  | 45+ | 3.20 (1.99 to 5.15) | 2.87 (1.57 to 5.24) | <0.001 |
| EF category | |  |  |  |
|  | >=50% | 1.76 (1.36 to 2.28) | 1.00 |  |
|  | 41 to 49% | 1.45 (0.60 to 3.49) | 0.83 (0.33 to 2.06) | 0.680 |
|  | <=40% | 1.53 (0.69 to 3.41) | 0.87 (0.38 to 2.02) | 0.746 |

| **Table 6: Cox bivariable regression controlling for current age and each variable in turn for the association between DOAC choice and stroke rate among people taking DOACs for stroke prevention in NVAF.** | | | | | | |
| --- | --- | --- | --- | --- | --- | --- |
|  |  | **Apixaban** | **Rivaroxaban** |  | **Dabigatran** |  |
|  |  | ***HR*** | ***HR (95% CI)*** | ***p-value*** | ***HR (95% CI)*** | ***p-value*** |
| Unadjusted | | 1.00 | 0.66 (0.36 to 1.18) | 0.160 | 0.90 (0.47 to 1.73) | 0.742 |
| Sex | | 1.00 | 0.65 (0.36 to 1.17) | 0.149 | 0.96 (0.49 to 1.85) | 0.896 |
| Dose given | | 1.00 | 0.66 (0.37 to 1.20) | 0.173 | 0.89 (0.46 to 1.72) | 0.740 |
| Dose indicated | | 1.00 | 0.62 (0.34 to 1.13) | 0.115 | 0.91 (0.47 to 1.75) | 0.773 |
| Creatinine clearance | | 1.00 | 0.67 (0.37 to 0.96) | 0.185 | 0.96 (0.50 to 1.86) | 0.907 |
| COPD | | 1.00 | 0.67 (0.39 to 1.20) | 0.179 | 0.88 (0.46 to 1.69) | 0.702 |
| Weight category | | 1.00 | 0.65 (0.36 to 1.18) | 0.156 | 0.92 (0.48 to 1.78) | 0.156 |
| RVSP | | 1.00 | 0.68 (0.38 to 1.24) | 0.209 | 0.94 (0.48 to 1.81) | 0.846 |
| HLD | | 1.00 | 0.64 (0.36 to 1.15) | 0.138 | 0.90 (0.46 to 1.73) | 0.742 |
| Atrial flutter | | 1.00 | 0.67 (0.37 to 1.21) | 0.185 | 0.93 (0.48 to 1.79) | 0.823 |
| Paroxysmal afib | | 1.00 | 0.68 (0.38 to 1.23) | 0.204 | 0.96 (0.49 to 1.87) | 0.899 |
| DM | | 1.00 | 0.66 (0.36 to 1.18) | 0.159 | 0.89 (0.46 to 1.72) | 0.737 |
